# EMT-led laryngeal tube vs. face-mask ventilation during cardiopulmonary resuscitation - a multicenter prospective randomized trial

**DOI:** 10.1186/s13049-017-0446-1

**Published:** 2017-10-26

**Authors:** Anna Fiala, Wolfgang Lederer, Agnes Neumayr, Tamara Egger, Sabrina Neururer, Ernst Toferer, Michael Baubin, Peter Paal

**Affiliations:** 1 Department of Anesthesiology and Critical Care Medicine, Medical University of Innsbruck, Anichstrasse 35, 6020 Innsbruck, Austria; 2 Medical University of Innsbruck, Anichstrasse 35, 6020 Innsbruck, Austria; 3 Department of Medical Statistics, Informatics and Health Economics, Medical University of Innsbruck, Anichstrasse 35, 6020 Innsbruck, Austria; 4 Department of Anaesthesiology and Intensive Care Medicine, Hospitallers Brothers Hospital, Teaching Hospital of the Paracelsus Private Medical University Salzburg, Salzburg, Austria; 5 Barts Heart Centre, William Harvey Research Institute, Barts & The London School of Medicine & Dentistry, Queen Mary University of London, London, UK

**Keywords:** Airway management, Cardiac arrest, Cardiopulmonary resuscitation, Laryngeal tube, Prehospital emergency medicine

## Abstract

**Background:**

Laryngeal tube (LT) application by rescue personnel as an alternate airway during the early stages of out-of-hospital cardiac arrest (OHCA) is still subject of debate. We evaluated ease of handling and efficacy of ventilation administered by emergency medical technicians (EMTs) using LT and bag-valve-mask (BVM) during cardiopulmonary resuscitation of patients with OHCA.

**Methods:**

An open prospective randomized multicenter study was conducted at six emergency medical services centers over 18 months. Patients in OHCA initially resuscitated by EMTs were enrolled. Ease of handling (LT insertion, tight seal) and efficacy of ventilation (chest rises visibly, no air leak) with LT and BVM were subjectively assessed by EMTs during pre-study training and by the attending emergency physician on the scene. Outcome and frequency of complications were compared.

**Results:**

Of 97 eligible patients, 78 were enrolled. During pre-study training EMTs rated efficacy of ventilation with LT higher than with BVM (66.7% vs. 36.2%, *p* = 0.022), but efficacy of on-site ventilation did not differ between the two groups (71.4% vs. 58.5%, *p* = 0.686). Frequency of complications (11.4% vs. 19.5%, *p* = 0.961) did not differ between the two groups.

**Conclusions:**

EMTs preferred LT ventilation to BVM ventilation during pre-study training, but on-site there was no difference with regard to efficacy, ventilation safety, or outcome. The results indicate that LT ventilation by EMTs during OHCA is not superior to BVM and cannot substitute for BVM training. We assume that the main benefit of the LT is the provision of an alternative airway when BVM ventilation fails. Training in BVM ventilation remains paramount in EMT apprenticeship and cannot be substituted by LT ventilation.

**Trial registration:**

ClinicalTrials.gov (NCT01718795).

**Electronic supplementary material:**

The online version of this article (10.1186/s13049-017-0446-1) contains supplementary material, which is available to authorized users.

## Background

Supraglottic airways including the laryngeal tube (LT) enable rapid and effective ventilation in most cases [1]. Contrarily, conventional bag-valve-mask (BVM) ventilation and endotracheal intubation may be difficult, especially when caregivers have little experience.

The promoted simplicity in handling makes the LT an attractive device for airway management during cardiopulmonary resuscitation (CPR), even for healthcare providers with only basic training [2]. Success rates after short training on manikins were reported between 72% and 94% in emergency medical technicians (EMTs) [1, 3–5]. Kurola et al. observed that the LT may enable rapid and effective airway control as compared to BVM when used by inexperienced personnel [6]. The quick insertion of the LT may result in shorter hands-off intervals, increased chest compression fraction [7] and may consequently improve chest compression quality [8]. Muller et al. observed that mean tidal volume and mean minute volume were higher with LT ventilation than with BVM ventilation [8]. Ventilation by LT may be particularly advantageous when anatomic conditions, e.g. facial hair, edentulism, facial dysmorphia and obesity, make BVM ventilation difficult or even impossible.

Application of the LT by trained EMTs during CPR has been legal in Austria since 2010. We aimed to investigate subjectively assessed ease of handling (LT insertion, tight seal) and efficacy of ventilation (chest rises visibly, no air leak) with LT as compared to BVM ventilation as performed by EMTs after pre-study training and during CPR in OHCA.

## Methods

### Study design

The Institutional Review Board of the Medical University of Innsbruck approved this open prospective randomized multicenter study, which was conducted from September 2012 to February 2014. Airway management with EMT-led LT vs. BVM ventilation during out-of-hospital cardiac arrest (OHCA) was investigated in six physician-staffed emergency medical services (EMS) centers in Tyrol, Austria. In the case of presumed OHCA reported to the dispatch center, the ambulance and physician-staffed EMS closest to the emergency site were simultaneously dispatched. Due to the high density of ambulances EMTs frequently arrived on the scene first and provided basic life support until the emergency physician arrived. Only patients initially resuscitated by EMTs who completed their pre-study training were randomly assigned to airway management with either laryngeal tube suction – disposable (LTS-D, VBM Medizintechnik GmbH, Sulz a.N., Germany) or BVM (AMBU Spur II by Ambu A/S, Baltorpbakken 13, Ballerup, Denmark). All ambulances in the catchment area were equipped with an opaque envelope attached to each airway management set containing information on the randomization order. On scene, EMTs started basic life support (BLS, i.e. chest compression, ventilation according to randomization, and defibrillation if indicated) [2]. During BLS, chest compression and ventilation were continued at a ratio of 30:2 in both groups [2]. Efficacy of the EMT-guided ventilation was evaluated by the emergency physician as soon as he arrived at the scene by determining whether the chest rises visibly after each inflation without air leak. Data were recorded with mobile medical devices (Corpuls3, software ed.2.3, YOM 2011, G.Stemple GmbH, 86,916 Kaufering, Germany).

Inclusion criteria were: OHCA in patients ≥18 years of age. Exclusion criteria were: lack of consent of the involved EMT and/or emergency physician, emergency physician arriving at scene and starting airway management prior to arrival of the EMT, presumed airway obstruction, death of the patient before EMS arrival. It was agreed that if two attempts failed, the mode of airway management would be changed to the alternate ventilation technique. The study was designed according to intention to treat. A study manager regularly observed completeness of equipment and documentation.

### Pre-study training

LT training followed the manufacturer’s recommendations (http://www.vbm-medical.de/cms/files/a5-1.0_06.08-de%2D-web%2D-.pdf). Similarly, BMV training was conducted according to international CPR guidelines [2]. Three months before study commencement, 203 EMTs completed a 2-h training session in LT insertion and ventilation, and a refresher course in BVM ventilation on manikins (Resusci Anne Advanced Skilltrainer CE, 151–20,033, YOM 2011, Laerdal Medical, 4002 Stavanger, Norway) at the Red Cross Academy in Innsbruck, Austria. At least three successful LT insertions with consequent sufficient ventilation (i.e. chest rises visibly after each ventilation without relevant air leak, evaluation performed by an emergency physician) were required to pass the training course.

### Data collection

The data spread sheet was composed according to the Utstein Style Guidelines for OHCA [9], and the CONSORT 2010 guidelines [10]. Data collection was jointly performed by the attending EMT, the pre-hospital emergency physician, and the admitting hospital physician (Additional file 1). The EMT arriving first at the scene assessed quality of bystander CPR (location, depth and frequency of chest compressions, and whether ventilation was performed or not). The EMT recorded initial cardiac rhythm, interval between arrival on site and adequate ventilation, interval between CA (if witnessed) and arrival of EMT, interval between onset of CPR and arrival of emergency physicians (Additional file 1).

During pre-study training ventilation efficacy was subjectively assessed by EMTs using an on-line questionnaire (www.2ask.at; amundis Communications GmbH, Felix-Wankel-Str. 4, Constance, Germany). The primary study end-points were ease of handling and efficacy of ventilation assessed by EMTs. Secondary study end-points included ventilation attempts, efficacy of ventilation assessed by emergency physicians, and complications (Additional file 1).

### Statistical analysis

The assumed null hypothesis for the primary study end-points was that ease of handling and efficacy of ventilation do not differ between LT and BVM ventilation. The sample size was calculated for an alpha-error of 0.05 and a power of 80% (beta-error of 0.2) to detect significant efficacy of ventilation in the LT group. A minimum of 25 applications in each group was deemed sufficient according to evaluation of the pre-study training. Categorical data were reported as frequencies and compared using the chi-square test. Ordinal data were reported as median and were analyzed using the Mann–Whitney U test or Spearman-Rho correlations. Results were deemed significant with a *p* value <0.05.

## Results

### Pre-study training assessment

All participating EMTs completed the questionnaire after training. Efficacy of LT ventilation was rated successful by most (66.7%) and regarded as more efficient (*p* = 0.022) than BVM ventilation (Table 1). According to the EMTs’ subjective assessment, ease of handling correlated with efficiency of ventilation when using the LT (*p* = 0.037). Ventilation problems were reported frequently in both groups (LT 44.4% vs. BVM 48.3%, *p* = 0.695). 86.1% of EMTs considered their LT training to be sufficient; 13.9% would have preferred additional training.

**Table 1:** Subjective EMT assessment of efficacy and ease of handling for LT and BVM ventilation after pre-study training using a 10-point scale regarding efficacy (1 = very low, 10 = very high) and ease of handling (1 = impossible, 10 = very easy)

	LT group (*n* = 54)	BVM group (*n* = 58)	*p*-value
Efficacy, credits (n; %)			0.022
1	14 (25.9)	13 (22.4)	
2	1 (1.9)	7 (12.1)	
3	0	4 (6.9)	
4	1 (1.9)	3 (5.2)	
5	0	2 (3.4)	
6	1 (1.9)	3 (5.2)	
7	0	5 (8.6)	
8	1 (1.9)	10 (17.2)	
9	6 (11.1)	5 (8.6)	
10	29 (53.7)	6 (10.3)	
Ease of handling, credits (n; %)			0.171
1	4 (7.4)	7 (12.1)	
2	2 (3.7)	1 (1.7)	
3	4 (7.4)	3 (5.2)	
4	0	4 (6.9)	
5	2 (3.7)	8 (13.8)	
6	0	7 (12.1)	
7	0	5 (8.6)	
8	8 (14.8)	10 (17.2)	
9	8 (14.8)	7 (12.1)	
10	24 (44.4)	5 (8.6)	

### On-site assessment

**Fig. 1:**
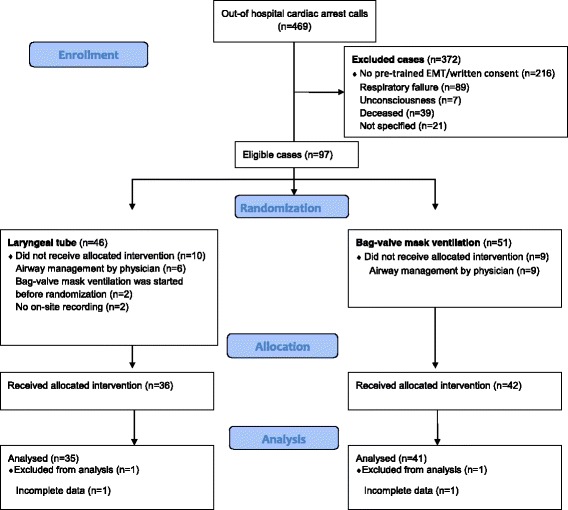
Flow Diagram (Consort 2010) of patient enrolment, randomization, allocation, and analysis

During the study period 469 calls of presumed OHCA (i.e. unresponsive person, no detectable breathing) were reported to the dispatch centre. 372 patients were not eligible to randomization (in 216 cases advanced life support was started either with EMTs lacking LT pre-training and/or with lacking EMT written consent). Ninety-seven cases were randomized (randomization rate 20.7%), and ultimately 78 patients included (inclusion rate 80.4%). Two patients were excluded because of incomplete data. Thus, 35 (46.1%) patients were ultimately allocated to the LT group and 41 (53.9%) to the BVM group (Fig. 1). There were no significant differences in patient characteristics or OHCA findings between the two groups (Table 2). In 26 (74.3%) patients the LT was successfully inserted and positioned on the first attempt. Efficient ventilation was confirmed by the attending emergency physician (LT 71.4% vs. BVM 58.5%, *p* = 0.686). We noted a tendency to lower oxygen saturation (first measurement after ROSC) with BVM ventilation. ROSC occurred in 16 patients (21.1%).

**Table 2:** Patient characteristics and cardiac arrest findings on-site in thirty-five patients with laryngeal tube ventilation and in forty-one patients with bag valve mask ventilation

	LT group (*n* = 35)	BVM group (*n* = 41)	*p*-value
Patient characteristics			
Male gender (n; %)	23; 65.7	26; 63.4	0.811
Age (year; SD)	69.1 ± 17.4	71.4 ± 13.7	0.554
Witnessed arrest (n; %)	11; 31.4	15; 36.6	0.993
Hospital discharge (n; %)	1; 2.9	1; 2.4	0.848
Intervals			
Call - CPR onset (median; IQR)	3 (1; 9.5)	4 (1; 7)	0.885
Call - effective ventilation (min; ±SD)	10.1 ± 8.0	8.9 ± 5.8	0.705
Call - hospital arrival (min; ±SD)	68.4 + 50.5	53.1 + 13.3	0.953
Basic Life Support			
Bystander CPR (n; %)	18; 51.4	13; 31.7	0.169
Effective CPR (n; %	11; 31.4	6; 14.6	0.095
Advanced Life Support			
Effective ventilation (n; %)	25; 71.4	24; 58.5	0.686
Tracheal intubation (n; %)	11; 31.4	9; 22.0	0.374
First CO_2_ (mm Hg; SD)	33.0 ± 16.9	23.5 ± 19.6	0.12
First documented ECG rhythm			0.606
Asystole (n; %)	20; 57.1	17; 41.5	
Pulse-less electrical activity (n; %)	6; 17.1	7; 17.0	
pVT/VF (n; %)	8; 22.9	11; 26.8	
ROSC (n; %)	9; 25.7	7; 17.1	0.478
Heart rate (mean, ±SD)	87.2 ± 23.1	73.0 ± 38.6	0.375
Systolic blood pressure (mean, ±SD)	122.4 ± 39.2	94.6 ± 28.8	0.185
O_2_ saturation (mean, ±SD)	91.1 ± 7.9	86.8 ± 8.7	0.272
Complications			0.961
Aspiration (n)	0	1	
Airway bleeding (n)	1	1	
Regurgitation (n)	4	7	

*BVM* bag valve mask, *CO*_*2*_ carbon dioxide, *CPR*cardiopulmonary resuscitation, *ECG* Electrocardiography, *IQR interquartile range, LT* laryngeal tube, *n* number, *O*_*2*_ oxygen, *pVT* pulseless ventricular tachycardia, *ROSC*return of spontaneous circulation, *SD* standard deviation, *VF* ventricular fibrillation

On-site complications comprised aspiration in one patient (BVM group) and injuries to the mucosal membrane indicated by blood stain on the device, one in the LT group and one in the BVM group.

## Discussion

EMTs preferred LT ventilation over BVM ventilation in the pre-study training, but on-site assessment regarding ease of handling and efficacy, frequency of complications and outcome showed no differences between the two methods. We prospectively studied ease of handling and efficacy of LT and BVM ventilation performed by EMTs during pre-study training. The standardized training program allowed comparison of LT and BVM airway management and ventilation in real life OHCA patients. However, data acquisition was prone to incomplete recording as compared to findings of previous experimental studies. As simulated conditions may substantially differ from real CPR situations, our prospective study design allowed subjective assessment of ventilation by EMTs after pre-study training and objective evaluation of ventilation by emergency physicians during real OHCA.

After training, 66.7% of EMTs in our study appraised LT ventilation as being highly efficient. This corresponds with findings made in other studies of LT ventilation administered by EMTs and nurses showing success rates between 72 and 94% [1, 3–5, 11]. Although most EMTs in our study had only basic experience (fewer than ten LT insertions), they more often cited good ease of handling and fewer problems as compared to BVM. Roth et al. reported that LT ventilation in real CPR was more successful than BVM ventilation (93% vs. 30%) [1]. In our study the attending emergency physicians confirmed efficient ventilation by EMTs in cases for LT (71.4%) as well as for BVM (58.5%; *p* = 0.686). Presumably, the pre-study refresher in BVM ventilation may have had an impact on the frequency of efficient BVM ventilation.

After training, EMTs regarded LT ventilation as superior to BVM ventilation; only 13.9% of EMTs considered additional training with LT insertion and ventilation necessary. However, EMTs frequently reported difficulties with ventilation in both groups during pre-study training. Sunde et al. observed a high number of insertion-related problems with LT ventilation [12]. The authors concluded that promising results in manikin studies may not be applicable to real-life CPR [12]. We assume that high expectations for the LT may create a subjective reality. Perceptions of advantage and disadvantage may influence performance and efficacy beliefs in a competitive situation [13]. Applied to our pre-study results this would mean that expectations of EMTs for the LT may eventually lead them to behave and achieve in ways that confirm their expectations.

In most patients on site, effective ventilation was provided within the first 10 min of OHCA. Within this interval airway management is not expected to substantially influence outcome. Iwami et al. reported that in patients with CA of presumed cardiac origin chest compression only (and defibrillation, if indicated) is superior to combined respiratory and cardiac resuscitation within the first 5 min of CPR [14]. Maignan et al. compared 41 cases with intermittent chest compressions in the BVM group to 41 cases with continuous chest compressions in the LT group. Airway management with the LT was associated with a 27% increase in the chest compression fraction and significantly reduced hands-off intervals but survival to discharge did not differ significantly between the two groups [7]. We doubt that increased chest compression fraction can be achieved with the comparatively low LT leak pressure. An estimated leak pressure of approximately 36 cm H_2_O was reported for LT ventilation [15]. Therefore, in our study intermittent chest compression and ventilation were continued at a ratio of 30:2 after LT insertion as we expected low LT leak pressure to interfere with continuous chest compression and simultaneous ventilation.

We encountered only one airway bleeding (blood stain on the device) and no case of aspiration in the LT group. However, factors associated with unsuccessful LT ventilation in the prehospital setting are numerous including incorrect placement of the tube in the trachea or in the pharynx, mucosa swelling of the tongue and throat and unrecognized airway obstruction [7, 16, 17]. Incorrect LT placement may cause gastric inflation, regurgitation and massive pulmonary aspiration. Dengler et al. recommended that LTS should be used in all cases of emergency airway management [16].

Tanabe et al. reported in a nation-wide study that prehospital use of supraglottic airway devices was associated with poorer neurological outcome as compared to tracheal intubation [18]. Results from animal research indicate that carotid blood flow in the low-perfusion state during CPR is further diminished by pressure on the carotid arteries from inflated LT cuffs [19].

The time may be nearing when BVM ventilation will lose its prominence as the standard ventilation technique during basic life support in favor of supraglottic airway devices [20]. However, LT ventilation during cardiac arrest is not a strikingly simple solution. Currently, training in BVM ventilation remains paramount in EMT apprenticeship.

Limitations of our study arise from the fact that the study was conducted in a selected sample of OHCA patients collected from six different centers. The study design determined the enrollment of cases with OHCA, where one of 203 trained EMTs had started CPR and airway management before arrival of the emergency physician. This offers considerable risk of a selection bias as EMTs without training were not allowed to participate in the study, and whenever the emergency physician arrived first he initiated ALS airway management. As EMTs do not intubate OHCA patients in our county, we did not evaluate tracheal intubation by EMTs for efficacy and ease of handling. Of the EMTs 13.9% would have preferred additional training after the pre-study training. Procedural bias from anticipated pressure to perform might have induced some of the EMTs to not participate. Correlations between mode of ventilation and survival to discharge were not calculated as we do not know the various clinical aspects that might have influenced the outcome.

## Conclusions

EMTs preferred LT ventilation to BVM ventilation during pre-study training, but on site no difference was seen in efficacy, ventilation safety or outcome. The results indicate that LT ventilation by EMTs during OHCA is not superior to BVM ventilation and that LT cannot substitute for BVM training. We assume that the main benefit of the LT is the provision of an alternative airway when BVM ventilation fails. Training in BVM ventilation remains paramount in EMT apprenticeship and cannot be substituted by LT ventilation.

## Additional file

Additional file 1: Data collection form. (DOCX 29 kb)

## Abbreviations

BVM: Bag valve mask
CPR: Cardiopulmonary resuscitation
ECG: Electrocardiography
EMS: Emergency medical services
EMT: Emergency medical technicians
ERC: European Resuscitation Council
LT: Laryngeal tube
OHCA: Out-of-hospital cardiac arrest
pVT: Pulseless ventricular tachycardia
ROSC: Return of spontaneous circulation
VF: Ventricular fibrillation
