# Supplementary material for: EMT-led laryngeal tube vs. face-mask ventilation during cardiopulmonary resuscitation - a multicenter prospective randomized trial
Source: Scand J Trauma Resusc Emerg Med. 2017 Oct 26;25:104. doi: 10.1186/s13049-017-0446-1 (PMC5658918; doi:10.1186/s13049-017-0446-1)
Supplement: Additional file 1: — Data collection form. (DOCX 29 kb) [file 13049_2017_446_MOESM1_ESM.docx]

**Additional file 1. Data collection form**

Randomization:

- LT
- BVM

**1) Data acquisition by EMT**

| Grade of training | - EMT - EMT with training in drug administration - EMT with training in intravenous access - EMT with training in tracheal intubation |
| --- | --- |
| Cardiac arrest | - Witnessed - Not witnessed |
| Quality of CPR by first responder | - Sufficient - Not sufficient - Chest compression only - Automated external defibrillation by layperson - Chest compression and ventilation - No CPR - Additional notes:….. |
| Initial cardiac rhythm | - Cardiac rhythm with signs of circulation - Ventricular fibrillation (VF)/pulseless ventricular tachycardia (pVT) - Pulseless electrical activity (PEA) - Asystole |
| Airway management by first responder | - Bag-valve mask ventilation (BVM) - Oro-pharyngeal airway - Mouth-to-mouth ventilation - None - Other: …… |
| First responder is | - Layperson - General practitioner - Nursing personnel - EMT - Other: ….. |
| Estimated interval between “picking up airway device” until first ventilation | - Min:sec ….:…. |
| Sufficient ventilation (chest rises visibly) | - Yes - No |
| Interval between collapse and start of CPR | - Min:sec ….:…. |
| Interval between CPR onset and arrival of emergency physician | - Min:sec ….:…. |
| Attempts of airway management by EMT | - LT: one attempt - LT: two attempts - BVM: one attempt - BVM: two attempts - More than two attempts - Change of device: from LT to BVM - Change of device: from BVM to LT |
| Transport of patient | - With ambulance - Airborne - Name of hospital: …… - No transport |

**2) Data acquisition by emergency physician**

| Co-morbitities | - Healthy patient - Minor co-morbitity - Major co-morbitity - Major co-morbitity with constant danger to life - No presumed survival without operation within 24hrs - Others: …. - Not known |
| --- | --- |
| Age of patient | - …. Years - Not known |
| Gender | - Male - Female - Pregnant: Yes / no |
| Type of emergency | - Trauma - No trauma |
| Cardiac arrest | - Not witnessed - Witnessed by layperson - Witnessed by EMT |
| Initial cardiac rhythm at arrival of emergency physician | - Cardiac rhythm with signs of circulation - VF/pVT - PEA - Asystole |
| Sufficient airway management by EMT (chest rises visibly, no air leak) | - Yes - No - Additional notes: …. |
| Sufficient chest compressions  (location, depth and frequency of chest compressions) | - Yes - No - Not |
| End-tidal CO_2_ (mmHg) | - First measurement: ….. - Highest: ….. - Lowest: ….. |
| Suction via LT | - Yes: …… - No |
| Regurgitation with | - BVM - LT |
| Changing from LT to tracheal tube | - Yes - No - If yes, why: ….. |
| Changing from BVM to tracheal tube | - Yes - No - If yes, why: ….. |
| Successful tracheal intubation | - Yes - No |
| Outcome | - Heart rate after ROSC: …../min - Systolic blood pressure after ROSC (mmHg): ….. - SpO_2_ after ROSC (%):….. - Prehospital ROSC - Death on site - Other: ….. |
| ECG at the end of CPR | - Rhythm with spontaneous circulation (e.g. sinus rhythm, atrial fibrillation) - VF/pVT - PEA - Asystole - Other: ….. |
| ECG at hospital arrival | - Rhythm with spontaneous circulation (e.g. sinus rhythm, atrial fibrillation) - VF/pVT - PEA - Asystole - Other: ….. |
| Intervals | - From collapse to arrival of EMT: …..min - From collapse to CPR onset: ….. min - From collapse to efficient ventilation: …… min - From collapse to hospital arrival: …..min |

**3) Data acquisition by attending physician at hospital:**

| Initial systolic blood pressure after arrival | …… mmHg |
| --- | --- |
| Initial SaO_2_ after arrival | ….. % |
| Initial etCO_2_ after arrival | …… mmHg |
| Aspiration (verified by bronchoscopy within 24hrs after arrival) | - Yes - No |
| Pneumonia during stay at hospital | - Yes - No - if yes, what day (after arrival): ….. |
| Survival to discharge from hospital | - Yes - No |
| Survival 28 days after CPR | - Yes - No - Unknown |
| Cerebral performance category (CPC) at discharge from hospital | - 1 - 2 - 3 - 4 - 5 |
| Severe adverse events (SAE) | - Malposition of LT - Dental trauma - Regurgitation prior to airway management - Regurgitation during airway management |
| Bleeding | - Minimal - Moderate - Severe - Where: ….. |
| Other SAE | …… |
| Specify | …… |
| What measures were taken? | …… |
| Is there causal relationship between SAE and airway management? | - Yes - No - Uncertain |
| Complications during transport | - Yes - No - If yes, specify: ….. |
| Consequences of SAE | - Recovery - Recovery with impairment - Not yet recovered - Death, causally determined |
